# Physical activity monitoring-based interventions in geriatric patients: a scoping review on intervention components and clinical applicability

**DOI:** 10.1186/s11556-023-00320-9

**Published:** 2023-05-18

**Authors:** Rieke Trumpf, Laura Elani Schulte, Henning Schroeder, Rasmus Tolstrup Larsen, Peter Haussermann, Wiebren Zijlstra, Tim Fleiner

**Affiliations:** 1 Institute of Movement and Sport Gerontology, German Sport University Cologne, Am Sportpark Muengersdorf 6, 50933 Cologne, Germany; 2 Department of Geriatric Psychiatry & Psychotherapy, LVR Hospital Cologne, Cologne, Germany; 3 Department of Public Health, Section of Social Medicine, University of Copenhagen, Copenhagen, Denmark; 4 Department of Occupational- and Physiotherapy, Copenhagen University Hospital, Rigshospitalet, Copenhagen, Denmark; 5 Institute for Geriatric Research, Ulm University Medical Center, Ulm, Germany

**Keywords:** Physical activity tracker, Wearables, Older adults, Medical condition, Personalized treatment

## Abstract

**Objective:**

To identify and analyze the components applied in interventions using physical activity (PA) monitoring in geriatric patients and determine their feasibility and applicability.

**Methods:**

A systematic search in six databases (PubMed, Embase, SPORTDiscus, CINAHL, Web of Science, and GeroLit) was conducted to identify studies reporting interventions that included the application of a PA monitor in adults aged ≥ 60 years with a clinical diagnosis. PA monitor interventions were analyzed regarding their feedback, goal-setting and behavior change technique (BCT) components. To determine the feasibility and applicability of interventions, the participants’ adherence to the intervention, their experience as well as adverse events were analyzed.

**Results:**

Seventeen eligible studies, applying 22 interventions, were identified. Studies included a total of 827 older patients with a median age of 70.2 years. In thirteen interventions (59%), the PA monitor was embedded in a structured behavioral intervention, an indication-specific intervention or usual care. Most frequently applied intervention components were goal setting and self-monitoring (*n* = 18), real-time PA monitor feedback complemented by feedback from the study team (*n* = 12), use of further BCTs (*n* = 18), and regular counseling with the study team (*n* = 19). Comprehensive information on the participants’ intervention adherence and experience were reported for 15 (68%) and 8 (36%) interventions, respectively.

**Conclusion:**

The components included in PA monitoring-based interventions varied considerably especially regarding the extent, frequency, and content of feedback, goal setting and BCTs counseling. Future research should evaluate which components are most effective and clinically applicable to promote physical activity in geriatric patients. To be able to precisely analyze the effects, trials should seek to report details on intervention components, adherence and adverse events, while future reviews may use the findings of this scoping review to conduct analyses with less heterogeneity in study characteristics and intervention strategies.

## Introduction

Regular physical activity (PA) is a key aspect in the prevention and management of chronic diseases and functional decline in aging [1]. A poor health status is one of the most important determinants of and self-perceived barrier to physical activity in older adults [2, 3]. Only 40 to 55% of older adults meet the World Health Organization’s (WHO) guidelines on PA, recommending 150–300 min of moderate-to-vigorous aerobic intensity PA throughout a week for substantial health benefits [4]. To prevent progress of disease and disability and thus, preserve independency and health-related quality of life, there is an urgent need for clinically applicable strategies to monitor and effective interventions to promote PA over the continuum of care [5]. These strategies and interventions should be tailored to older adults’ individual capability and needs.

Deriving such interventions requires assessment tools that provide reliable and detailed information on habitual PA levels. Historically, recall questionnaires and activity logs have been used to assess PA, however, the estimation of PA levels based on self-report methods are susceptible to several biases [6]. Over the past decade, objective assessment methods including body-worn PA monitors, such as accelerometers and inertial measurement units have become the primary choice to monitor PA. Simultaneously, the rapid advances of information and communication technologies have brought a plethora of consumer grade wearable devices (e.g., pedometers, fitness tracker, and smart watches) to the market. PA monitors can generate various parameters that provide an objective feedback on PA (e.g., number of steps). Their use has been associated with increased physical activity levels [7]. Besides objective feedback on PA, PA monitors promote several behavior change techniques (BCT) such as self-monitoring and goal setting that are frequently used in life-style interventions to facilitate behavioral changes [8–10]. The individual tailoring of PA goals and using real-time PA data monitoring throughout an intervention are important features especially in a population (i.e. older patients) not meeting the PA recommendations [3, 10].

Research on the application of PA monitors to assess and intervene on PA is growing rapidly. Literature reviews indicated a moderate effectiveness of body-worn PA monitors to promote PA in (older) adults [11–17]. Previous reviews have defined no or rather broad inclusion criteria regarding the intervention components, leading to a diversity in intervention strategies [17]. Especially in health care, the PA monitors are often used in combination with other BCT components such as psychoeducation on the positive effects of being active, and behavioral counselling including goal setting and identification of barriers or they are embedded into usual care which in turn often contains BCT components [13, 16, 17]. Further methodological heterogeneity arises from differences in the PA monitor devices [13] and in the frequency, extent, and delivery mode of feedback on PA [17].

Given this methodological heterogeneity, there are currently few reviews that can report consistently on the effectiveness of PA monitor-based interventions in geriatric patients. Better knowledge on the applied intervention components is required to be able to conduct consistently focused reviews on the effects of PA monitor-based interventions in geriatric patients and to identify promising intervention approaches for clinical application. For the latter, aspects concerning the feasibility of interventions are also of interest, such as adverse events, as well as the participants’ adherence and experience with the intervention [18].

Therefore, the objective of this scoping review is to identify and analyze the components applied in PA monitoring based interventions in geriatric patients. We seek to determine their feasibility in order to identify promising intervention approaches and to guide a way towards consistently focused research on the effects of interventions using PA monitoring in geriatric patients. This review aims to identify and analyze the following components of interventions: (1) the PA monitors applied (2) whether the PA monitor component was used in combination with other interventions (e.g., indication-specific, behavioral, usual care), (3) the frequency, extent, and delivery mode of feedback on PA (4) whether and how PA goals were personalized based on PA data from the PA monitor, and (5) the BCT components applied. In order to determine the feasibility and clinical applicability of interventions, adverse events as well as the participants’ adherence to and experience with the intervention were analyzed.

## Methods

A protocol of this scoping review has been registered in the PROSPERO database (CRD42020203954). The reporting has been conducted according to the PRISMA extension for scoping reviews [19].

### Eligibility criteria

The eligibility criteria were specified according to the PCC (participants, concept, context) approach for scoping reviews [20].

#### Participants

Studies enrolling participants aged ≥ 60 years and with a confirmed clinical diagnosis of any medical condition according to the International Statistical Classification of Diseases (ICD- 10) [21] or the Diagnostic and Statistical Manual of Mental Disorders (DSM-5) [22] were eligible.

#### Concept

Studies were included if the intervention included any kind of PA monitor (e.g., pedometer, accelerometers, or smartphones) and participants received objective feedback on their PA (e.g., number of steps) based on data from the PA monitor. Studies were excluded if no outcomes on PA were reported.

#### Context

No in-/exclusion criteria regarding cultural/sub-cultural factors, geographic location, specific racial as well as gender-based interests or a specific setting were applied.

The language of included studies had to be English or German, thus studies reporting in any other language were excluded. No restriction of publication date was applied.

### Search methods for identification of studies

A systematic search strategy was developed using preliminary searches and relevant publications. Relevant keywords and MeSH/ Thesaurus terms were identified to delimit (1) the population of interest and (2) the PAM intervention and (3) the outcome targeted by the intervention. Finally, the search strategy covered a combination of the following keywords and related terms for: ‘geriatrics’, ‘activity tracker’, ‘physical activity’ and ‘health-related outcomes’. The full search strategy can be found in the appendix.

The final systematic search was conducted on May 1^st^, 2022. The following databases were searched: PubMed, Embase, SPORTDiscus, CINAHL, Web of Science, and GeroLit. Additional studies were obtained by hand searching the reference lists of relevant reviews. Furthermore, international experts in the field of research were contacted and asked to recommend additional articles and ongoing projects they knew and would fit the research question.

### Study selection and data extraction

Identified studies were imported into rayyan, a web and mobile app for collaborative work on systematic reviews [23]. Three authors (HS, LS & RT) screened titles and abstracts independently. Disagreements were solved by discussion before full-text assessments. Screening of full texts and data extraction were performed independently by two authors (LS & RT). Diverging assessments were solved by discussion with the last author (TF).

The following data items were extracted: author, year of publication, country, sample characteristics (sample size, age, clinical diagnosis and setting) and intervention components based on the template for intervention description and replication (TIDieR) checklist [24]. If one study included two or more PA monitor- based intervention arms, we checked if participants of both arms received any kind of feedback based on data from the PA monitor. If so, data were extracted for all intervention arms to which this applied. BCTs were assessed based on the taxonomy of behavior change techniques by Abraham and Michie [25].

To determine the feasibility and applicability of the PA monitor-based interventions, information on adverse events and qualitative feedback on the participants’ adherence (e.g., adherence to sensor usage and compliance with PA goals) and experience with the intervention were extracted.

### Analysis

In order to be able to compare the applied interventions, their components were grouped into categories as shown in Table 1 and the frequency of interventions was assessed for each component. Similarities in the components of included interventions were investigated based on the UpSet plot analysis [26]. The UpSet plot analysis employs a scalable matrix-based visualization to show intersections of data sets and their size [27]. It was generated using the UpSetR package [27] in RStudio 1.4.1106 for macOS [28]. The implementation of the intervention components as well as results regarding the participants’ adherence, experience, and adverse events were analyzed in a narrative review.

**Table 1:** Overview of categorization of intervention components

**Component**		**Yes/No**
**Intervention**	PA monitor as main intervention component	
	Additional to usual care, indication-specific intervention (e.g., weight-loss program) or structured behavioral intervention	
**Device**	Pedometer (limited to the assessment of steps during walking)	
	PA monitor (enable to assess other activities) Consumer grade device Research grade device	
	Use of corresponding application or web platform	
**Main PA target**	Steps per day	
	time of walking/light intensity PA per day	
	Sedentary time per day	
**Goal- setting**	Fixed goals (e.g., 7.000 steps/day)	
	Based on individual PA data (e.g., baseline step count)	
	Goal- setting standardization	
	Tailoring during the intervention	
**Self-monitoring**	Using PA monitor	
**Feedback**	By PA monitor/application only	
	Additional feedback provided	
	Feedback only provided by coach	
	Frequency (daily, (≤ once per week, > once per week)	
**BCTs**	Use of other BCT components besides feedback, goal setting and self-monitoring	
**BCT counseling**	Individual or group-based	
	Mediation mode (face-to-face, telephone or other)	
	Frequency (≤ once per week, > once per week)	

*BCT* behavior change technique, *PA* physical activity

## Results

**Fig. 1:**
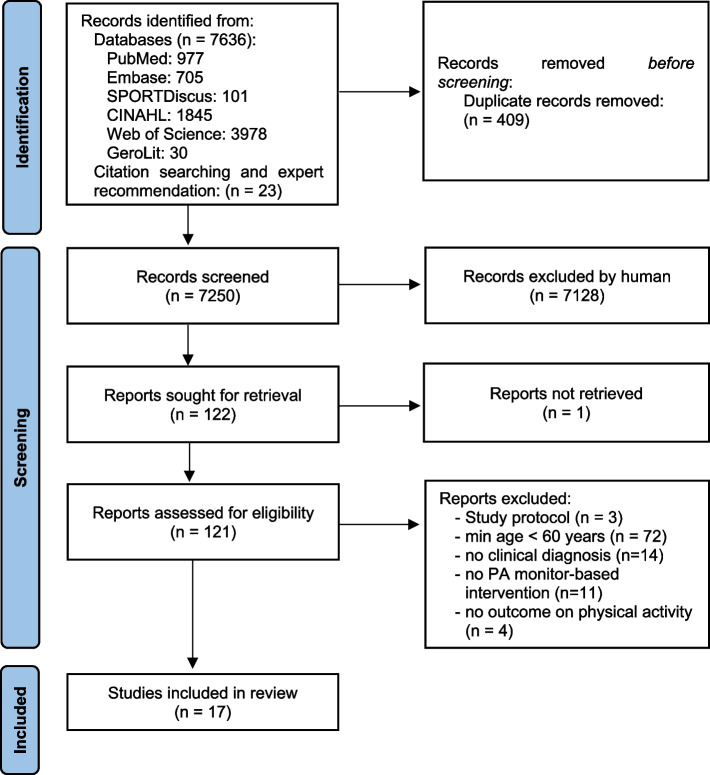
Flow of study selection process

Seventeen studies were included [29–45]. The study selection is illustrated in Fig. 1.

### Characteristics of included studies

A summary of the included studies is provided in Table 2. The majority of the included studies (*n* = 13, 77%) were published within the past five years. Eleven of the selected studies were RCTs [29, 31–33, 35, 37–39, 42, 45, 46] and six were non-randomized intervention trials [30, 34, 36, 40, 43, 44].

**Table 2:** Summary of the characteristics of included studies (*n* = 17)

Characteristic	Number of studies (%)
**Design**	
RCT with parallel group design [29, 31–33, 35, 37–39, 41, 42, 45]	11 (65)
Non-randomized study of intervention [30, 34, 36, 40, 43, 44]	6 (35)
**Participant diagnoses**	
Cancer survivors [42, 45]	2 (12)
Chronic heart failure [36]	1 (6)
Chronic kidney disease [31]	1 (6)
Chronic Obstructive Pulmonary Disease [33, 43]	2 (12)
Kidney transplant recipients [41]	1 (6)
Obesity [35, 38]	2 (12)
Osteoarthritis [29, 39, 40]	3 (18)
Mild Cognitive Impairment [44]	1 (6)
Morbus Parkinson [30]	1 (6)
More than one clinical indication [32, 34, 37]	3 (18)
**Setting**	
Community-dwelling [30, 32, 33, 36, 38–42, 44, 45]	11 (65)
Outpatient [31, 34]	2 (12)
Inpatient rehabilitation/hospital [29, 37, 43]	3 (18)
No information [35]	1 (6)
**Participant characteristics**	**Median (range)**
Median sample size	34 (10 to 270)
Median age in studies	70.2 (64 to 81.5)
Median body mass index in studies [29, 31, 33–39, 42, 43]	28.4 (21.9 to 35.4)
Median percentage of male participants in studies	44.0 (5 to 100)
Median baseline daily step count [29–31, 34–36, 38–43, 45]	5016 (3034 to 9516)

*RCT* Randomized Controlled Trial. The reported median of mean values is unweighted in relation to study size or reporting precision

The total number of participants was 827, however, the studies varied considerably with regard to the number and characteristics of participants included. The median sample size was 34 participants per study. Peel and colleagues (2016) included the highest number of participants (*n* = 270). The majority of the studies (*n* = 14, 82%) included participants with a specific clinical diagnosis [29–31, 33, 35, 36, 38–40, 42–46]. The trials included patients with osteoarthritis [29, 39, 40], obesity [35, 38], chronic obstructive pulmonary disease (COPD) [33, 43], Morbus Parkinson [30], chronic kidney disease [31], chronic heart failure [36], kidney transplant recipients [41], and mild cognitive impairment [44]. The participants of two studies were cancer survivors [42, 45]. Three studies did not focus on a specific indication but included patients with various medical conditions [32, 34, 37]. In eleven studies, a home-based intervention in community-dwelling patients was implemented [30, 32, 33, 36, 38–42, 44, 45]. In two studies, the PA monitor-based intervention was conducted in an outpatient setting [31, 34] and three studied conducted the intervention during inpatient treatment [29, 37, 43]. The setting was not clearly specified in one study [35].

### Intervention components

The intervention components used in each of the included studies are presented in Table 3. Four studies included two relevant intervention arms that were included as separate interventions [31, 34, 41, 42, 45]. Hence, a total of 22 interventions were included in the analysis. Table 4 shows the frequency of intervention components used in the included studies.

**Table 3:** Intervention characteristics of included studies

	Intervention strategies				BCT components				Goal				
					Feedback			Self-mnt	Setting		Other BCTs		
Ref	Components	PA monitor	App	Parameter	How	Mode	Frequency of delivery		Overall	Tailoring	Components	Mode	Frequency of delivery
Talbot et al. 2003 [39]	PA monitor	New Lifestyles Digi-walker SW-200	no	steps	real time + stud.team	face-to-face	weekly	applied	30% ↑ of steps/day from baseline	10% ↑ of steps/day every 4 weeks	FP	face-to-face	monthly
Nicklas et al. 2014 [35]	PA monitor + behav.int + ISI	Lifecorder Plus	no	walking time	real time + stud.team	face-to-face	weekly	applied	20% ↑ in volume of light PA from baseline	initially 10% ↑ in volume of light PA, further ↑ throughout the intervention	BI + CP + RM + GE + SC + FP	face-to-face	weekly
Hiraki et al. 2017 [31]	PA monitor	Kenz Lifecorder EX	no	steps	real time + stud.team	face-to-face	bi-monthly	applied	8000–1000 steps/day	not applied	not applied	na	na
Hiraki et al. 2017 [31]	PA monitor	Kenz Lifecorder EX	no	steps	real time	na	na	applied	not applied	not applied	not applied	na	na
Brandes et al. 2018 [29]	PA monitor + usual care	Step Activity Monitor 3.0	no	steps	stud.team	face-to-face	twice in a week	not applied	not applied	5% ↑ of steps/day compared to previous 3–4 days	CP + GE + SI + SC + FP	face-to-face	2x/week
Rosenberg et al. 2020 [38]	PA monitor + behav.int	Jawbone UPband + ActivPal	no	sedentary time	real time + stud.team	face-to-face	tri-weekly	not applied	60 min ↓ sitting time per day	individually	BI + CP + SC + MI + GE + SI	face-to-face&Telephone	tri-weekly
Peel et al. 2016 [37]	PA monitor + usual care	ActivPal	no	walking time	stud.team	face-to-face	daily	not applied	not applied	individually	GE + SC + FP	face-to-face	weekly
Janevic et al., 2020 [32]	PA monitor	Fitbit Zip	yes	steps	real time + app	na	na	applied	not applied	not applied	not applied	na	na
Kawagoshi et al. 2015 [33]	PA monitor + ISI	Lifecorder Ex	no	steps	real time + stud.team	face-to-face	monthly	applied	8000 steps/day	not applied	SM	face-to-face	monthly
Pinto et al. 2021 [45]	PA monitor	Fitbit Charge 2	yes	steps	real time + stud.team + app	message	bi-weekly	applied	not applied	2000 steps/week:20%↑ 3000 steps/week:15%↑ 4000 steps/week:10%↑ > 5000 steps/week:5%↑	CP + SI + RW + SC	app	by choice
Pinto et al. 2021 [45]	PA monitor	Fitbit Charge 2	yes	steps	real time + stud.team + app	message	bi-weekly	applied	not applied	2000 steps/week:20%↑ 3000 steps/week:15%↑ 4000 steps/week:10%↑ > 5000steps/week:5%↑	SI + RW + SC	app	by choice
O’Brien et al. 2021 [41]	PA monitor + behav.int	Fitbit Charge 2	yes	steps	real time + app	na	na	applied	not applied	individually	E + BI + CP + SC + TM + GE + SI + FP	group face-to-face	monthly
O’Brien et al. 2021 [41]	PA monitor	Fitbit Charge 2	yes	steps	real time + app	na	na	applied	not applied	not applied	E + SC + FP	group face-to-face	monthly
Blair et al. 2021 [42]	PA monitor	Jawbone Up 2	yes	sedentary time	real time + app	na	na	applied	↓ sedentary time by ↑ of light PA	↑ of > 3000 steps/day from baseline	E + CP + SI	telephone	twice
Blair et al. 2021 [42]	PA monitor + behav.int	Jawbone Up 2	yes	sedentary time	real time + app	na	na	applied	↓ sedentary time by ↑ of light PA	↑ of > 3000 steps/day from baseline	E + BI + CP + SC + GE + SI	telephone	monthly
Morey, et al. 2019	PA monitor	Wrist-worn PA tracker	no	steps	real time + stud.team	Telephone	weekly	applied	not applied	↑ of > 100 steps/day from to baseline	E + GE	telephone	weekly
Morey, et al. 2019	PA monitor	Wrist-worn PA tracker	no	steps	real time	na	na	applied	not applied	not applied	not applied	na	na
Colón- Semenza et al. 2018 [30]	PA monitor + behav.int	FitBit Zip	yes	steps	real time + stud.team + app	face-to-face	weekly	applied	not applied	individually	E + BI + SC + MI + GE + SI + RW + SC	face-to-face	weekly
Okwose et al. 2019 [36]	PA monitor + behav.int	Omron Health care	no	steps	real time + stud.team	Telephone	weekly	applied	↑ of 2000 steps/day from baseline	not applied	BI + CP + SC + GE + SI	telephone	weekly
Zaslavsky et al. 2019 [40]	PA monitor + behav.int	Fitbit Charge 2	yes	steps	real time + stud.team + + app	message	weekly	applied	not applied	not applied	CP + RM + MI + SI + RW + FP	telephone	not specified
Nickerson et al. 2021 [44]	PA monitor	Whithings Activé	yes	steps	real time + stud.team	not specified	not specified	applied	not applied	not applied	SC + TM + GE	telephone	bi-weekly
Wshah et al. 2022 [43]	PA monitor + usual care + behav.int	activPAL	no	sedentary time	stud.team	face-to-face	weekly	not applied	↓ sedentary time by ↑ of light PA	individually	E + BI + CP + SC + ST + TM + GE + SI + FP	face-to-face	once

*app* application, *BCT* Behavioral change technique, *behave.int* structured behavioral intervention, *BI* Barrier identification, *CP* Use of cues and prompts, *E* Education, *FP* Follow-up prompts, *GE* general encouragement, *ISI* Indication-specific intervention, *MI* motivational interviewing, *na* not applicable, *PA* physical activity, *RM* Role model, *RW* Rewards, *SC* Social support/ comparison, *Self-mnt* Self monitoring, *SI* Specific instruction, *SM* Stress management, *ST* Self-talk, *stud.team* study team, *TM* Time management, ↑ Increase, ↓ Decrease

**Table 4:** Frequency of the components used in the interventions (*n* = 22) based on the Template for intervention description and replication (TIDieR)

TIdieR Items		Intervention components	Frequency in interventions (%)	
Materials		**Device**		
		Pedometer [29–33, 35, 36, 39]	9	(41)
		Physical activity monitor [34, 37, 38, 40–45]	13	(59)
		Use of device-corresponding application [30, 32, 40–42, 44, 45]	10	(45)
Procedure		**Intervention components**		
		Physical activity monitor as main intervention component [31, 32, 34, 39, 41, 42, 44, 45]	9	(41)
		Embedded in usual care [29, 37, 43]	3	(14)
		Combined with an indication-specific intervention [33, 35]	2	(9)
		Combined with a structured behavioral intervention [30, 35, 36, 38, 40–42]	8	(36)
		**Target parameter**		
		daily number of steps [29–34, 36, 39–41, 44, 45]	16	(73)
		daily walking time/ light physical activity intensity [35, 37]	2	(9)
		daily time spent sedentary [38, 42, 43]	4	(18)
	Tailoring	**Goal setting** [29–31, 33–39, 41–43, 45]	**16**	**(73)**
		Individualized [29, 30, 34–37, 39, 41–43, 45]	13	(59)
		Tailored during the intervention [29, 30, 34, 35, 37–39, 41–43, 45]	13	(59)
	How	**Feedback on physical activity**		
		only real-time from physical activity monitor/ application [31, 32, 34, 41, 42]	7	(32)
		Physical activity monitor/application & feedback from study team [30, 31, 33–36, 38–40, 44, 45]	12	(55)
		Only from study team [29, 37, 43]	3	(14)
	How much	**Frequency of feedback from study team**		
		Daily [37]	1	(5)
		≥ once per week [30, 31, 33–36, 38–40, 43, 45]	12	(55)
		< once per week [29]	1	(5)
		Not specified [44]	1	(5)
		**Self-monitoring using physical activity monitor** [30–36, 39–42, 44, 45]	**18**	**(82)**
		**Use of other behavior change techniques** [29, 30, 33–45]	**18**	**(82)**
		Barrier identification [30, 35, 36, 38, 41–43]	7	(32)
		Use of cues and prompts [29, 35, 36, 38, 40–43, 45]	10	(45)
		Education [30, 34, 41–43]	7	(32)
		Use of follow-up prompts [39–41, 43]	5	(23)
		General encouragement [29, 30, 34–37, 41–44]	10	(45)
		Motivational interviewing [30, 38, 40]	3	(14)
		Role model [35, 40]	2	(9)
		Rewards [30, 40, 45]	4	(18)
		Social support/ comparison [29, 35–38, 41–45]	13	(59)
		Specific instruction [29, 30, 36, 38, 40–42, 45]	11	(50)
		Stress management [33]	1	(5)
		Self-talk [43]	1	(5)
		Time management [41, 43, 44]	3	(14)
		**Mode of behavior change technique mediation** [29, 30, 33–40, 42–45]	**18**	**(82)**
	How	Individual [29, 30, 33–40, 42–45]	16	(73)
		Group-based [41]	2	(9)
		Face-to-face [29, 33, 35, 37–39, 41, 43]	10	(45)
		Telephone [34, 36, 38, 40, 42, 44]	7	(32)
		only via application [45]	2	(9)
	How much	**Frequency of behavior change technique mediation**		
		≥ once per week [30, 33–44]	14	(64)
		< once per week [29]	1	(5)
		by choice [45]	2	(9)
		Not specified [40]	1	(5)
	who	**Behavior change technique mediated by**		
		Study team [29, 34–36, 38, 40–44]	12	(55)
		Healthcare staff [33, 37, 43]	3	(14)
		Peer group [30]	1	(5)
		Application [45]	2	(9)
	where	**Setting**		
		Home-based [30–33, 36, 39–42, 44, 45]	18	(82)
		Hospital/ rehabilitation clinic [29, 37]	3	(14)
		not specified [35]	1	(5)

#### PA monitor-based intervention component

In nine interventions a body-worn PA monitor and BCTs facilitated by the PA monitor (i.e., feedback, goalsetting, self-monitoring) were used as main intervention strategy [31, 32, 34, 39, 41, 42, 44, 45], whereas in 13 interventions the PA monitor-based component was embedded into usual care, an indication-specific intervention and/or combined it with a structured behavioral intervention [29, 30, 33–38, 40–43, 45]. Nine interventions used a pedometer [29–33, 35, 36, 39] and 13 interventions a PA monitor that was not limited to the assessment of walking activity [29, 34, 37, 38, 40–43, 45].

#### Feedback and self-monitoring

In 16 interventions, steps per day were used as feedback parameter [29–34, 36, 39–41, 44, 45]. Four interventions addressed sedentary time [38, 42, 43] and two studies walking time [35, 37]. In 19 interventions participants received real-time feedback from the body-worn PA monitor [30, 31, 33–36, 38–40, 44, 45]. The participants of two interventions, only received the real time feedback from the body-worn PA monitor [31, 34]. In ten interventions, participants were provided with access to device-associated software applications (e.g., Up by Jawbone™, Fitbit by Fitbit, Inc. or Withings Health Mate by Withings) [30, 32, 40–42, 44, 45], that complemented the real time feedback from the PA monitor. The participants of 12 interventions additionally received feedback from the study team [30, 31, 33–36, 38–40, 44, 45], mostly delivered face-to-face [30, 31, 33, 35, 38, 39]. The participants of three interventions had no access to real time feedback by the PA monitor or a corresponding application and received feedback on their PA only by the study team [29, 37, 43]. The frequency of feedback provided from the study team ranged from daily to monthly (Table 4).

Self-monitoring was applied in 18 interventions. In the three interventions that used research grade PA monitors (i.e., ActivPal without display), no self-monitoring was applied [29, 37, 43]. One intervention, using a consumer-grade PA monitor without display (i.e., Jawbone Up band), did not provide participants with access to the device-corresponding application and hence self-monitoring was not applied [38].

#### Goal setting

In 16 interventions, any kind of goal setting were used [29–31, 33–39, 41–43, 45]. In three interventions fixed overall goals were set, regardless of the results of the PA monitor measurements [31, 33, 38]. Participants of Hiraki and colleagues [31] as well as Kawagoshi and colleagues [33] were instructed to reach a step count goal of 8000 – 10,000 steps per day, while Rosenberg and colleagues [38] set the reduction of daily sedentary time by 60 min per day as overall goal for every participant. In six interventions the overall goal was individualized by adding a fixed number of steps [34, 36, 42] or a percentage [35, 39] to the participant’s baseline step count. In 13 interventions PA goals were tailored to the participants capability during the intervention [29, 30, 34, 35, 37–39, 41–43, 45]. In 8 of these 13 interventions, PA goals were tailored based on the data measured by the PA monitor by adding a certain percentage or number to the participant’s step count of the previous days [29, 35, 39, 45]. In five interventions PA goals were tailored individually by the study team [30, 37, 41].

#### Other BCT components

Besides feedback, self-monitoring, and goal setting, the following BCT components were identified (listed by frequency in descending order): social support and/ or comparison, specific instructions, use of cues and prompts, general encouragement, barrier identification, education, use of follow-up prompts, rewards, time management, motivational interviewing, identification as a role model, stress management and self-talk. In 18 interventions one or more of these BCT components were used (Table 3) [29, 30, 33–45]. Four interventions did not use BCT components [31, 32, 34]

### Similarities of the interventions

The upset plot analysis of similarities of the interventions revealed a variety of component combinations between the interventions. Thirteen interventions combined the following components: regular counseling by the study team (e.g., for feedback on PA and/or BCT delivery), use of other BCTs than goal setting, feedback and self-monitoring, goal setting, and feedback on PA from the study team [29, 30, 33–39, 41–43]. In three out of these interventions, the PA monitor was used as the main intervention component [34, 39, 42]. In all three of these interventions individualized goals were applied. Two out of the three interventions applied face-to-face contacts to deliver feedback and/or BCTs [34, 39]. Ten interventions embedded the PA monitor component into another intervention (e.g., usual care) [29, 30, 33, 35–38, 41–43]. Seven out of these ten interventions applied individualized PA goals [30, 35–37, 41, 42] and seven interventions delivered feedback and/or BCTs face-to-face [29, 33, 37, 38, 41, 43]. Four out of the seven interventions used both individualized PA goals and face-to-face contacts to deliver feedback and/or BCTs [29, 35, 37, 41].

### Participants’ adherence and experience, adverse events

For most interventions (*n* = 15, 68%) information on adherence to PA monitor usage were reported [29, 33, 35, 37, 39–45]. The median percentage of days participants wore the PA monitor device reported for 9 interventions was 87% [29, 33, 35, 39, 41, 44, 45], ranging from 57% [29] to 99%[44]. Wear times of the PA monitor was reported for 4 interventions [29, 35, 37, 40]. The median wear time per day was 11.5 h per day and ranged from 8.3 h per day [37] to more than 20 h per day [40]. In one study (*n* = 2 interventions) the PA monitor device and application usage were evaluated based on the participants’ self-report using the 5-point Likert scale [42]. All participants (*n* = 29) agreed or strongly agreed to have worn the PA monitor (Jawbone Up) on most days of the week, however, 62% of the participants indicated that they ignored the alert from the device and remained seated when reminded to stand up and move [42].

Information on achievement of set PA goals were provided for 4 interventions [33, 35, 39, 43]. The median percentage of days on which PA goals were met was 57%, ranging from 48 to 81%. Wshah and colleagues [43] reported that 73 goals were set over the intervention period, of which 41 (56%) were met.

Information about the occurrence or non-occurrence of adverse events were available for 8 interventions [30, 31, 36, 38, 42]. Only Rosenberg and colleagues [38] reported that 10% of the participants experienced mild skin irritation from the PA monitor device (Jawbone Up band).

Participants’ satisfaction with the intervention components was analyzed in 8 studies [30, 32, 38, 42, 43, 45]. The median proportion of participants who were satisfied with the overall intervention strategy was 89%, ranging from 89% [45] to 92% [38] and was reported for four interventions [38, 43, 45]. Participants’ satisfaction with the PA monitor device was reported for four interventions [32, 38, 42]. The median proportion of participants who were satisfied with the PA monitor usage was 79% and ranged from 79% [38, 42] to 96% [32]. Problems with the PA monitor device were reported for three interventions [30, 32, 38]. Twenty-five percent of the participants in the intervention by Janevic and colleagues [32] had problems to synchronize the device (Fitbit Zip) with the corresponding application. One participant (2%) in the intervention of Colón-Semenza and colleagues [30] reported problems in handling the PA monitor device (Fitbit Zip). Four participants (9%) of the intervention described Rosenberg and colleagues [38] experienced the PA monitor (Jawbone UP band) as not helpful and two participants (7%) reported that they did not use the PA monitor. Participants of three interventions were asked, if wearing the PA monitor device made them more aware of their PA level [32, 45]. The reported agreement ratios ranged from 41% [45] to 75% [32]. Participants of three interventions were asked if they would continue to use the PA monitor after the intervention ended [32, 42]. The agreement ratios ranged from 57% [32] to 79% [42].

## Discussion

The objective of this scoping review was to identify and analyze the components applied in PA monitoring based interventions in geriatric patients and to assess their feasibility and clinical applicability.

### Summary of evidence

In this scoping review we identified 22 interventions in which PA monitors were applied to provide geriatric patients with objective feedback on their PA levels. Our results revealed that the PA monitors were most frequently combined with structured behavioral health interventions, an indication-specific intervention or usual care. Most of the interventions focused on the daily number of steps as target parameter for self-monitoring, feedback and/or goalsetting. Other most frequently applied intervention components were goal setting, adjunct feedback from the study team, the use of further BCTs, and regular counseling with the study team. More than half of the included interventions combined all of the former four components. Despite the overlap in the use of these intervention components, we found differences in their implementation, which will be addressed in the following discussion considering the available findings on feasibility and applicability of the different approaches.

### PA monitor and feedback component

A wide range of different PA monitors were applied which vary in their ability to measure PA parameters and to provide corresponding feedback. In 41% (*n* = 9) of the interventions simple pedometers were applied. Pedometers measure walking activity and provide information on related parameters, such as the number of steps (most frequently used) or walking time and distance. Hence, they are essential to programs that recommend a specific step count goal or requiring self-monitoring of daily steps taken. However, the traditional devices (e.g., KENZ Lifecoder EX) often do not enable automatic data transmission, requiring users to manually transcribe data to activity logs which limits their applicability for long-term PA monitoring. Furthermore, the lacking accuracy of simple pedometers in the assessment of steps often lead to overestimations in step counts, which might induce higher effect sizes when compared to accelerometer-based PA monitors [13]. Lacking accuracy is also one reason indicated by older adults preventing the use in their daily lives [47]. Although the present review identified the lowest adherence rates for research grade devices [29, 37], data on the adherence to PA monitor usage were often not reported for pedometer-based interventions [30–32, 36]. In order to fully understand the benefits of PA monitors and to estimate the applicability of single devices, future studies should report consistently on the adherence to PA monitor usage and any barriers leading to non-adherence.

Although pedometers are considered well accepted by older adults because they are usually easier to operate, participants aged more than 60 years also appear to be receptive to using more sophisticated PA monitors and learn to use them quite easily [47]. Such devices, allowing the assessment of other activities not limited to walking and also enabling the assessment of sedentary behavior, were used in 13 interventions (59%). The detailed assessment of physical activity enables to provide users with more comprehensive feedback on health enhancing/ threatening PA behaviors not limited to walking. However, the accuracy of corresponding assessment methods as well as the access to feedback and its delivery mode differ between devices. In three interventions participants wore a research grade PA monitor (e.g., ActivPal), which does not enable to provide the wearer with real-time feedback on PA. Hence, the participants received feedback on their performance only at times when it was provided to them by the study team. On the contrary, patients who received a modern consumer grade PA monitor, i.e., Fitbit Zip or Charge 2, Jawbone UP, Whithings Activé, received detailed real-time feedback on their PA and also had (except in one intervention) access to a software application at their convenience. These applications often provide even more detailed and interactive visualized feedback on various parameters related to PA [10], even those that are not necessarily part of the intervention. Extended feedback (e.g., number of calories burned) might additionally motivate to increase PA; however, the amount and complexity of health-related information can make it difficult for users to understand and interpret the data, leading to feelings of overwhelm. With more activity trackers brought to the market and advances in their features, future studies are needed to investigate how the feedback component should be designed to effectively improve PA and sedentary behavior. Literature reviews should apply more specific inclusion criteria regarding the devices and their feedback options or conduct subgroup analyses for less methodological heterogeneity.

An important issue, that needs to be considered regarding commercially available PA monitors, is that information regarding their psychometric properties (e.g., validity and reliability) is often not available [48, 49] or it is unclear how they were assessed (i.e., was the validation performed in geriatric populations and under real-life conditions by independent parties?) [50]. Furthermore, the data processing and applied algorithms for PA analysis of consumer grade devices are often not accessible due to economic interests of the manufacturer [51]. Within this context, identifying non-wear times is an important aspect that affects all PA monitors in terms of their clinical applicability for assessing and intervening on PA in geriatric health care as misclassifications of non-wear times likely lead to an over or underestimation of PA levels [52].

Compared to recent research grade devices (e.g., the ActivPal), consumer-grade PA monitors enable the self-monitoring of PA, and the device-corresponding applications make it easy to share the objective information on PA, e.g., with institutions in the continuum of care, representing a promising solution for future health care using PA as a vital sign [53]. However, within this context enhancing the oversight of the wearable device industry, providing specific safety regulations to protect the privacy and security of personal data, and clarifying relevant medical responsibilities as well as rights between physicians and patients are crucial aspects that need to be addressed [54].

This might also be the reason why only Peel and colleagues [37] integrated the PA monitor component into the routine care process by discussing patient’s PA levels in the weekly case conference and providing patients and their therapists with daily feedback on PA measured with the ActivPal. None of the interventions conducted in in-patient settings [29, 37, 43] were designed to be continued in the follow-up treatment or to involve the outpatient treatment provider. At this point it is important to mention, that the health status of patients differ across the healthcare sectors (e.g., inpatient setting vs. community dwelling), complicating the implementation of interventions across the continuum of care [5]. However, PA monitors can provide objective feedback on PA (e.g., number of steps) that can be easily understood by the patients themselves and a multiprofessional treatment team across health care sectors. Future studies should investigate how the PA monitor and corresponding feedback could be implemented across the continuum of care.

### Personalization of PA goals

Of 16 interventions that used goal setting, 12 interventions applied personalized PA goals that were based on the data from the PA monitor. Especially in geriatric health care, where patients are prone to fail the general PA recommendations, it is important to set measurable, attainable goals [3] and to monitor progress carefully [37]. The continuously assessed data from the PA monitors enables the former and further allows to set goals in line with the patient’s previous/current performance and ability level, which have been shown to be important aspects to improve physical activity engagement [55]. However, only less than half of the interventions (8, 44%) used the opportunity to adapt PA goals during the intervention based on the continuous PA data. Furthermore, the personalization approaches ranged from standardized procedures (e.g., adding a percentage of steps to the number of steps per day) [29, 35, 45] to individualized goals without any further details on the goal setting process [30, 37, 41]. Replicating the interventions and applying them in clinical practice requires more detailed information on how PA goals were personalized. Future research should aim to improve the personalized goal setting and to evaluate their effects.

Besides individualizing the amount and volume of PA goals, personally tailored advice regarding its timing and environmental aspects could further help to improve the intervention adherence [56]. Personalized timing of interventions was realized by using the real-time data from the PA monitor within three interventions [38, 42]. In all three interventions the overall aim was to reduce the time spent sedentary using Jawbone Up band and its incorporated *idle alert* function to notify the user on inactivity via a gentle vibration of the wrist band after a user-specified time spent inactive. This offers the possibility to deliver the intervention when behaviors occur, that should be prevented – e.g. long periods of sitting time. Sometimes, however, external circumstances do not allow for immediate interruption or change in current behaviors. In all three interventions the alert was set to 15 min [38, 42]. The participants satisfaction with the PA monitor was lowest for the Jawbone UP band [38, 42]. In order to ensure the continued use of the PA monitors, the time limits should be set carefully and based on scientific findings or health guidelines. Furthermore, users could be given the opportunity to mute notifications for limited periods.

### BCT components

Eighty-two percent of the interventions (*n* = 18) included the use of one or more BCT components additionally to the BCTs promoted by the PA monitor (e.g., feedback on performance and self-monitoring), indicating the importance of combining the objective feedback from the PA monitor with BCTs. This is in line with the results of the review from Braakhuis and colleagues [17], who found combinations of one or multiple BCTs in all interventions using objective feedback from PA monitors. Besides feedback on performance and self-monitoring, goal setting, social- support and comparison, general encouragement, specific instructions how to change the behavior (e.g., reduce sedentary time by reducing the time watching TV) and the use of cues and prompts were revealed as BCT components considered important in the present interventions. However, the number of combined BCT components used in the present interventions as well as the frequency of their delivery varied considerably, ranging from none [31, 32, 34] to nine [43] and once during the intervention period [43] to twice per week [29], respectively. Unfortunately, not all interventions clearly indicated which BCT components were used and described their content sufficiently. Hence, BCTs could only be determined approximately making it difficult to draw clear conclusions regarding specific BCT components. It can be assumed that indication-specific interventions (e.g., weight loss programs in obesity) and usual care also incorporate the use of BCTs.

A recent meta-analysis found that neither the frequency of feedback from the PA monitor nor whether goal setting was applied influenced the effectiveness of PA monitors in adults < 65 years, but differences in the population characteristics [57]. The authors indicated that some patient populations (e.g., overweight participants or participants with depression or anxiety) might experience an ambiguous and even counterproductive influence from PA monitor feedback. Research also suggests that BCTs that are effective at increasing PA in younger adults may not be effective for older adults [58]. In order to be able to better understand how and which BCTs are relevant in PA monitor interventions for geriatric patients, future research needs to clearly indicate which BCTs are used and how they are applied (see Blair and colleagues [42] for a positive example).

Consumer grade PA monitors and corresponding software applications contain various BCT components [9, 10], however, the present and previous results [17] show, that they are usually delivered within in-person counseling. Using the BCTs incorporated in PA monitors and corresponding applications in combination with real-time tele-counseling can make behavior change interventions clinically applicable through conservation of resources and improved cost-effectiveness [9]. Further research is needed to determine the most effective intervention strategies, with regard to the amount and type of therapist contact and BCT components for specific patient populations.

The following limitations need to be addressed within this scoping review: Firstly, a conclusive assessment of promising intervention approaches with regard to their feasibility and clinical applicability could not be performed because information on the participants’ adherence to and experience with the intervention was rarely reported within the individual studies. Secondly, the number of BCT components applied and their content might not have been assessed completely accurate, as not all interventions clearly indicated in detail which access the participants had to BCTs that were incorporated in the PA monitors and the corresponding applications.

With further advances in the field of information and communication technologies and the popularization of personalized health concepts, wearable devices will inevitably play a greater role in the field of health care and become better integrated into daily lives [59]. The intervention components applied in older adults with chronical conditions so far differ clearly form each other and it seems that the potential of PA monitors with regard to the use of integrated BCTs components and PA-monitor data to personalize interventions has not been exploited to the fullest yet. Modern PA monitors enable the monitoring of activity behavior (physical activity and sedentary behavior) and also sleep continuously over 24 h/day. A more in-depth analysis of the latter two in terms of their interrelation could possibly enable to identify individual activity profiles [60, 61] that could be used to decide whether the participant’s intervention should focus on the improvement of PA or sedentary behavior.

## Conclusion

This scoping review gives an overview on the components applied in interventions using PA monitors to provide older adults in geriatric health care with objective feedback on their PA. The overall intervention strategies varied considerably especially regarding the implementation of the feedback and BCT components. Details on adherence and adverse events have often not been reported, limiting the determination of the interventions’ clinical applicability. Future research should focus on determining which intervention components are most effective in improving PA and especially sedentary behavior, investigate the effects of personalized PA goals and how PA-monitor based interventions can be applied over the continuum of geriatric health care. To be able to precisely analyze potential effects, trials should seek to report details on intervention components, particularly which BCTs are used and how they are applied, as well as details on adherence and adverse events. Future reviews may use the findings of this scoping review to conduct analyses with less heterogeneity in study characteristics and intervention components.

## Data Availability

Not applicable.

## Appendix

### PubMed search strategy

**Table:**

Participants	(("Aged"[Mesh]) OR (older adults[tiab]) OR (old adults[tiab]) OR (older adult[tiab]) OR (older people[tiab]) OR (frail elderly[tiab]) OR (senior*[tiab]) OR (elder*[tiab]) OR ("Aged, 80 and over"[Mesh]) OR ("Nursing Care"[Mesh]) OR (Geriatrics) OR ("Geriatric Nursing"[Mesh]) OR ("Frailty"[Mesh]) OR ("Above 60 years"[tiab]) OR (Hospital ward) OR (Clinic) OR (Acute Care) OR (outpatient clinic))
	*AND*
Intervention	(("Fitness Trackers"[Mesh]) OR ("Accelerometry"[Mesh]) OR (Activity Tracker*[tiab]) OR (Personal Fitness Tracker*[tiab]) OR (Physical Fitness Tacker*[tiab]) OR (monitor*[tiab]) OR (activity monitor*[tiab]) OR (accelerometer based tracker*[tiab]) OR (step monitor*[tiab]) OR (physical activity monitor*[tiab]) OR (step counter[tiab]) OR (pedometer[tiab]) OR (quantified movement[tiab]) OR (movement counter[tiab]) OR (hybrid PA monitor*[tiab]) OR (body worn PA monitor*[tiab]) OR (smartphone application[tiab]) OR (jawbone[tiab]) OR (vivoactive[tiab]) OR (tomtom[tiab]) OR (xiaomi mi band[tiab]) OR (moov now[tiab]) OR (misfit ray[tiab]) OR (nokia go[tiab]) OR (Fitbit[tiab]) OR (Yamax[tiab]) OR (Omron[tiab])) AND ((self reported physical activity[tiab]) OR (personal* activit*[tiab]) OR (individual* activit*[tiab]) OR (individual exercise[tiab]) OR (individual recommendation[tiab]) OR (personal recommendation[tiab]) OR (individual intervention[tiab]) OR (counseling[tiab]))
	*AND*
Outcomes	(("Quality of Life"[Mesh]) OR ("Exercise"[Mesh]) OR (physical activity[tiab]) OR (functional fitness[tiab]) OR (functional mobility) OR Steps[tiab] OR (steps per day[tiab]) OR pain[tiab] OR ("Activities of Daily Living"[Mesh]) OR ("Housing for the Elderly"[Mesh]) OR (moderate-to-vigorous physical activity) OR MVPA OR (Osteoarthritis) OR (arthroplasty) OR (arthritis) OR (joint replacement) OR (COPD) OR (chronic obstructive pulmonary disease) OR (Cardiac patients) OR (hypertension) OR (vascular diseases) OR (type 2 Diabetes) OR (impaired glucose tolerance) OR (intermittent claudication) OR (overweight) OR (obese) OR (dialysis) OR (breast cancer) OR (cancer) OR (neuromuscular disease) OR (stroke) OR (Parkinson disease) OR (impaired cognitive function) OR (intellectual difficulties) OR (fibromyalgia) OR (polyneuropathie) OR (PNP) OR (sarcopenia))
	*NOT*
*Exclusion of*	((healthy older adults) OR (child*) OR (animal) OR (adolescence) OR (young adult))
